# Education Research: Educating Child Neurology Residents About Psychogenic Nonepileptic Seizures: A Needs Assessment

**DOI:** 10.1212/NE9.0000000000200111

**Published:** 2023-12-28

**Authors:** Afsaneh Talai, Daniel A. Freedman, Dara V.F. Albert, for the Pediatric Epilepsy Research Consortium

**Affiliations:** From the Division of Pediatric Neurology (A.T.), Department of Pediatrics, University of Texas Southwestern Medical Center at Dallas and Children's Medical Center of Dallas; Department of Neurology (D.A.F.), Dell Medical School, Austin, TX; and Division of Child Neurology (D.V.F.A.), Nationwide Children's Hospital, Columbus, OH.

## Abstract

**Background and Objectives:**

Psychogenic nonepileptic seizures (PNES) are difficult to differentiate from epileptic seizures (ES) even for neurologists who see these conditions frequently. This difficulty is due to overlapping semiologic findings between the 2 diagnoses. Previous studies have shown that trainees, including neurology trainees, are not accurate in differentiating PNES from ES. Neurologists often find the communication of PNES difficult. Despite these challenges, most programs do not have formal curricula for teaching PNES, and there are no standards for residency curricula in this topic. The aim of this study was to understand the gaps in resident education on PNES.

**Methods:**

This study was accomplished through a needs assessment of current pediatric neurology residents and residency program directors (PDs). Two unique surveys were distributed, 1 for child neurology trainees and 1 for PDs. Questions were designed to understand trainees' self-reported knowledge, confidence, current education received, and desired teaching. Similarly for PDs, questions were designed to assess the state of education on PNES at their programs, sufficiency of education, and their desire for standardized curriculum.

**Results:**

Sixty-eight trainees and 21 PDs responded to the survey. Approximately one-quarter of trainees reported neutral to low levels of confidence and 38% reported neutral to low levels of knowledge in caring for patients with PNES. Trainees reported that directing patients with PNES to appropriate management was the most challenging aspect of care, followed by communicating the diagnosis, with 60% and 46% reporting difficulty, respectively. Only 21% of residents felt their current PNES education needs no improvement. One-fifth of PDs felt their current PNES education is not sufficient, and all reported they would incorporate a standardized curriculum. Trainees reported preferring to learn about this topic through lectures and simulation, while PDs preferred online modules and simulation.

**Discussion:**

While residents and PDs report high confidence and knowledge in treating pediatric patients with PNES, respondents felt improvement is needed to their curricula regarding this topic. Multiple learning methods are preferred, with emphasis on communicating the diagnosis and management of patients once the diagnosis has been made. PDs desire a standardized curriculum and would incorporate one into their programs. Findings of this study could be used to create a national curriculum.

## Introduction

Psychogenic nonepileptic seizures (PNES), also referred to as functional seizures, is a type of functional neurologic symptom disorder in which patients experience paroxysmal events of altered motor activity or consciousness, resembling that of epileptic seizures (ES). While ES are caused by misfiring of the brain that can be measured by EEG, PNES is thought to be related to biopsychosocial factors.^1^ Diagnosing and communicating the diagnosis of PNES is typically the responsibility of neurologists at most clinical sites.^2^ PNES is common, especially among adolescent patients, with a recent study finding the highest prevalence (60 in 100,000) in patients 15–19 years of age.^3^ However, PNES proves difficult to differentiate from ES given the overlapping semiologic findings, even for neurologists who diagnose these conditions frequently.^4-8^ This holds especially true for resident trainees, including neurology trainees.^4,9,10^ Furthermore, neurologists often find communicating the diagnosis of PNES challenging; objective differences are noted in the communication of PNES vs ES to patients.^11-13^ Despite these challenges, most child neurology programs do not have a formal curriculum for teaching PNES.^2,14^ Furthermore, there are no American Board of Psychiatry and Neurology standards for residency curricula for the management of PNES nor is it listed in the Accreditation Council for Graduate Medical Education (ACGME) program requirements or Child Neurology Milestones. Experts in the field have emphasized the importance of including education on functional neurologic disorders (FNDs) in graduate medical training.^15^

The aim of this study was to understand the perceived deficiencies in resident education on PNES through performing a needs assessment of current pediatric neurology resident trainees and residency program directors (PDs). Our first hypothesis is that resident trainees will express a lack of confidence and self-reported knowledge in caring for patients with PNES. Our second hypothesis is that PDs are not satisfied with the current level of education and will desire more structured curricula about PNES for resident trainees. This needs assessment may serve as the basis for creating a standardized curriculum for the education of pediatric neurology resident trainees on the topic of PNES.

## Methods

### Survey Design and Distribution

Two unique surveys were designed, 1 for resident trainees and 1 for PDs and associate PDs. Questions aimed at sampling the content were generated by consensus of experts in the field (A.T., D.A.F., and D.V.F.A.). For trainees, questions were designed to assess level of interest and level of confidence in managing patients with PNES, current level of education received, and desired style of teaching. Similarly for PDs, questions were designed to assess the current state of education on PNES at their respective programs, sufficiency of this education, and their desire for standardized curriculum on this topic. Questions that required self-reported evaluation used a 5-point Likert scale based on previous studies on optimal survey development.^16-19^ The final question allowed open-ended commentary on the education of PNES in residency. Questions were chosen specifically to assess the need for a standardized curriculum and the format in which such a curriculum should be developed. Study data were collected and managed using REDCap electronic data capture tools hosted at the University of Texas Southwestern (UTSW) Medical Center.

The survey was pilot tested for content and clarity with graduating postgraduate year (PGY)5 pediatric neurology residents; these residents would not have been eligible to participate in the study. Two residents each at Nationwide Children's Hospital and UTSW reviewed the survey. All residents reported that the questions were clear and easy to understand. Suggestions for additional questions were made by residents that were deemed to be beyond the topic in question. A resident suggested inclusion of “other” as an answer choice for certain questions, with the option to write in answers; this was incorporated into the survey. Author D.V.F.A., an associate PD, reviewed the survey questions for PDs to ensure clarity. See eAppendix 1 (links.lww.com/NE9/A58) for survey questions. The surveys were distributed to the following listservs: Pediatric Epilepsy Research Consortium (PERC), Professors and Educators of Child Neurology, Pediatric Neurology Coordinators Consortium, and American Epilepsy Society (AES). The surveys were also posted to 2 neurology-themed Facebook groups: Women Neurologists Group and Neurology Physicians Only. Four weeks later, email reminders were sent to a list of PDs obtained through the Child Neurology Society website, as well as to the PERC and AES listservs. Inclusion criteria included ACGME-accredited child neurology PDs, associate PDs, and child neurology residents. There were no exclusion criteria.

### Standard Protocol Approvals, Registrations, and Patient Consents

The study was reviewed and deemed exempt by the Institutional Review Board at UTSW because it involved “surveys, tests, interviews, or observations,” and specifically, the study recorded data in such a manner that the identity of human individuals could not be readily ascertained. Consent was waived as part of the exempt status.

### Statistical Analysis

Mixed methods were used to analyze the data. Descriptive statistics were used to analyze quantitative responses to questions. A qualitative approach was used to analyze responses to open-ended survey questions. Comparisons were made using independent sample *t* tests where appropriate between PDs and residents as well as between early and late trainees. Reliability testing of Likert-scale questions from each survey was performed by calculating the Cronbach α to assess internal consistency. Statistical analyses were performed by one of the authors (D.V.F.A.) using SPSS (IBM SPSS Statistics for Windows, Version 26.0, Released 2019; IBM Corp., Armonk, NY).

### Data Availability

Data not provided in the article because of space limitations may be shared (anonymized) at the request of any qualified investigator.

## Results

### Survey Distribution

The survey was sent via email to the aforementioned listservs, with a follow-up email sent 4 weeks later. We received 68 resident responses and 21 PD responses. The response rate was 8% for residents and 14% for program leadership based on 75 programs with 166 resident spots per year, assuming each program has a PD, an APD, and 5 years of residents who would have received the survey.

### Respondent Demographics

Resident level of training ranged from PGY1–7, with the greatest portion of respondents at the PGY5 level (29%). See Figure for distribution of residents' level of training. Programs represented by PDs ranged in size from 1 to 6 residents per year.

**Figure:**
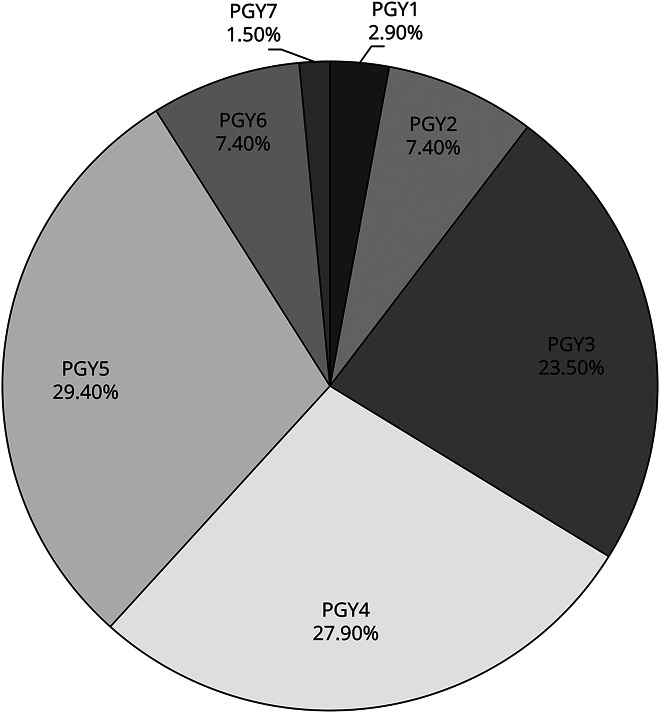
Distribution of Residents' Level of Training PGY = postgraduate year.

### Current Education Status

More than half of residents (56%) reported being interested in caring for patients with PNES. Residents reported receiving most of their PNES teaching through either didactic lectures (81%) or reading materials (54%). Similarly, PDs reported most education at their programs was given through didactic lectures (95%) or reading materials (62%). Online modules and standardized patient encounters represented less than 8% each of teaching received or given by residents and PDs, respectively. Of resident respondents, 12% reported receiving no formal teaching on PNES, and 5% of PDs reported having no formal curriculum. When asked about the sufficiency of the education they have received on PNES, 57% of residents felt their education is sufficient, whereas 81% of PDs felt the training they provide is sufficient (*p* = 0.549). All PDs reported wanting a standardized curriculum on this topic.

Seventy-five percent of all residents surveyed reported confidence in communicating the diagnosis of PNES to patients and families. When separated by level of training, 87% of senior residents (PGY 4–7) and 52% of junior residents (PGY 1–3) reported confidence in communicating the diagnosis (*p* = 0.002). Similarly, 86% of PDs reported confidence in their graduating residents' ability to communicate the diagnosis; when compared with all residents, no statistical difference was found (*p* = 0.862). Sixty-two percent of all residents reported feeling knowledgeable in their management of patients with PNES. When separated by level of training, 76% of senior residents and 39% of junior residents reported feeling knowledgeable (*p* = 0.001). Ninety-five percent of PDs reported confidence in their graduating residents' ability to diagnose and manage patients with PNES.

### Preferred Education

Residents reported, “communicating the diagnosis of PNES to patients/families” (46%) and “directing patients to appropriate treatment” (60%) were the most challenging aspects of caring for patients with PNES. When asked what format they would prefer for future education on this topic: didactics/lecture (54%) and simulation/standardized patients (41%) were the most popular formats requested. Reading materials (37%) and online modules (27%) were less preferred. Only 21% of residents felt their current PNES education needs no improvement. PDs reported wanting more teaching in the form of online modules (48%) and simulation/standardized patients (52%). Their least preferred formats were reading material (19%) and didactics/lectures (24%) (Table 1). PDs preferred either 2 or 3 hours for a dedicated curriculum, and only 14% were interested in a curriculum over 4 hours.

**Table 1:** Resident and PD Responses on the Status of Education on PNES and Preferred Type of Future Education on This Topic

	Resident responses (%)	PD responses (%)
Current education		
No formal PNES curriculum	12	5
Current education sufficient	57	81
Confidence in residents communicating the diagnosis	75	86
Preferred education		
Didactics/lecture	54	24
Simulation/standardized patients	41	52
Reading material	37	19
Online modules	27	48

Abbreviations: PD = program director; PNES = psychogenic nonepileptic seizure.

### Reliability Testing

The Cronbach α for the Likert-scale questions on the resident survey was 0.63 (modest) and the PD survey was 0.71 (acceptable).

### Narrative Responses

Twenty-nine narrative responses were provided by residents and 7 by PDs to optional open-ended questions. Two major themes were emphasized by both groups, including the management of patients with PNES as well as the importance of education on this topic (Table 2). Residents stated, “I think further mastery of the grounding techniques taught during the psychology sessions could be helpful for first line providers caring for this patient population” and “knowing where to refer patients and how to follow them long term would be very useful.” The importance of education was best illustrated by the following resident comment: “I think the more we educate trainees about these diagnoses from the beginning, the less frustration there will be for patients, and neurologists overall.” PDs also recognized the need for and importance of education on this topic, specifically mentioning “The main area that I think residents might most benefit is further skill development on speaking with patients and families about the diagnosis.”

**Table 2:** Narrative Responses Provided by Residents and PDs to an Optional Open-Ended Question on This Topic

Category	Subcategory	Description	Representative quote
Treatment and resource learning needs	Treatment	Desire to learn about treatment options for patients with PNES	I think further mastery of the grounding techniques taught during the psychology sessions could be helpful for first line providers caring for this patient population
	Resources	Need resources that are available for patients to learn more about their diagnosis	Having more workbooks, reading material and other supplements useful for patient education on PNES
More education	More education	More education on the topic is needed	I think the more we educate trainees about these diagnoses from the beginning, the less frustration there will be for patients, and neurologists overall
Other	Other	Understanding semiology, timing and type of testing to make diagnosis, communicating diagnosis	It would be good to review videos together with an experienced attending early in training and talk about what clues in the videos make one decide PNES vs seizure

Abbreviations: PD = program director; PNES = psychogenic nonepileptic seizure.

Treatment/resources refer to respondents' desire to learn about treatment options available to patients with PNES and resources that are available for patients to learn more about their diagnosis. Respondents also felt that more education on this topic is needed.

## Discussion

This study examined the learning needs of pediatric neurology residents in the education of PNES. The survey revealed that most of the residents feel confident in their ability to communicate the diagnosis, with that confidence increasing in the later years of training. This growth in confidence was also reflected in the knowledge residents have with PNES management, which significantly increased from juniors to seniors. This improvement seen with rise in rank is likely related to the growing experience residents have as they progress through residency. However, up to a quarter of graduating residents still do not feel knowledgeable in treating this patient population. PDs also reported a high level of confidence in their graduating residents' abilities to diagnose and communicate this diagnosis. While residents reported a high level of confidence and knowledge, most of them felt their programs' curricula could be improved, suggesting there is need for further refinement. Similarly, all PDs reported wanting a standardized curriculum on this topic, despite high confidence in their graduating residents' ability in managing patients with PNES. The difficulties with diagnosing and communicating PNES is consistently reported in the literature, both for residents and attending neurologists.^4-8,11,12^ The desire for further education by residents and PDs despite high levels of self-reported confidence therefore may be related to the sentiments reflected in the literature.

Nearly half of surveyed residents in our study reported not being interested in caring for patients with PNES. These findings are alarming in the context of a frequently encountered condition, with the estimated prevalence of PNES occurring in nearly 60 per 100,000 adolescents aged 15–19 years.^3^ It is possible this reported lack of interest is a reflection of gaps in knowledge, though biases regarding residents' interests within neurology and future fellowship training may be a factor.

There were additional notable findings in this study that emphasize the need for PNES-specific education in neurology residencies. A small but considerable proportion of residents reported not having any formal education on PNES. This is mirrored by similar data in adult neurology residencies in which a recent survey showed 18% of recently graduated residents did not receive formal instruction in PNES; as a result, there has been a call to develop a standardized curriculum for adult neurology residencies as well.^14^ Additionally, they reviewed existing resources from the International League Against Epilepsy (ILAE), AES, and the Functional Neurological Disorders Society (FNDS) for the PNES education of adult neurology residents. While the ILAE provides key learning points and the AES and FNDS webinars can serve as an essential starting point, a more developed standardized curriculum is needed in child neurology as it is in adult neurology.

Residents reported that most teaching they received was through didactics and reading, yet they prefer further education in the form of more didactics and simulation/standardized patient encounters. PDs similarly wanted curricula in the form of standardized patients, though they also wanted online modules, which was less preferred by residents. This may be explained by PDs' difficulty in balancing education and residents' numerous clinical duties; online modules may provide educational resources that residents can access outside of work hours. Albert et al. developed an Objective Structured Clinical Examination (OSCE) for a child neurology residency program that focuses on communication and includes a specific case of an adolescent with PNES. The case materials for the standardized encounter are published and could be used to incorporate a similar experience at other residency programs.^20^

PNES are a subtype of FNDs. Much like PNES, there is a paucity of data on FND in medical education. However, there are a few studies in other countries regarding undergraduate medical education or adult residency programs in psychiatry, neurology, and physiatry, which reflect similar findings as Milligan et al. and our survey results.^21-23^ Common themes included lack of FND-specific education and difficulties with the diagnosis. Researchers in Australia developed an “FND master class” focused on simulated conversation, which resulted in significant improvement in confidence regarding FND diagnosis and FND-related counseling.^22^

This survey highlights the need for a more robust and standardized curriculum for child neurology residents that emphasizes the modern conceptualization of PNES. A structured curriculum following the ILAE's outline with considerations of feasibility and attention to preferred learning methods would be ideal. Providing an assortment of learning methods could appeal to both residents and PDs, including didactics, online modules available through neurology organizations, and simulated patient encounters. Residents reported the most difficulty with communicating the diagnosis. We propose using role modeling, and where available, simulated conversations with standardized patients, as an evidence-based way of improving confidence with difficult conversations.^24-26^ Similar to Albert's OSCE curriculum, we recommend preconversation and postconversation assessments of competence to monitor the effectiveness of this curriculum.

Survey responses also indicated problems directing patients to appropriate treatment. This was further illustrated through narrative responses, with nearly half of responses pertaining to wanting more education on treatment and resources for patients with PNES. This desire for further education on the management of PNES is likely linked to the difficulty neurologists face in connecting patients with mental health providers and the lack of such providers who feel equipped to manage patients with PNES.^27^ Educating neurology residents on psychoeducation, resources, and further treatment options can help bridge the gap while their patients establish with mental health providers.

This survey-based needs assessment is limited by sample bias with a low response rate of 8% of all residents and 14% of all program leadership. This response rate, however, is comparable with a similar study conducted in adult neurology.^14^ The PD survey had an acceptable measure of internal consistency; however, the resident survey had a lower Cronbach α suggesting lower reliability.^28^ Lower correlation between question items on the resident survey may be because of a small sample size or heterogeneity of the questions (e.g., a resident might report a high level of interest but a low level of knowledge due to a deficit in education or exposure). A previously validated instrument was not available to assess these constructs. We attempted to have high content validity by generating questions via expert consensus and by piloting questions for clarity; however, it is possible the instrument does not fully evaluate the construct assessed due to the limited number of questions. Making the survey longer might have limited the responses. Unfortunately, measures of external validity were not possible given that there are no prior studies evaluating this content nor would it be feasible to compare responses on the survey to respondent behavior in the clinical or education settings. An additional limitation to this survey is not knowing how much exposure to epilepsy the respondents have, where they train, what their interests are within neurology, or what their intended career path is. The intended population for the survey was ACGME-accredited programs and thus should be limited to the United States. However, the use of Facebook neurology groups and AES listservs may have allowed residents or PDs from outside the United States, potentially confounding results.

While residents and PDs report high confidence and knowledge in treating pediatric patients with PNES, all respondents, residents, and program leadership alike feel improvement is needed to their curricula regarding this topic. Multiple learning methods are preferred, with emphasis on communicating the diagnosis and management of patients once the diagnosis has been made.

## Appendix 1 Authors

**Table:**

Name	Location	Contribution
Afsaneh Talai, MD	Division of Pediatric Neurology, Department of Pediatrics, University of Texas Southwestern Medical Center at Dallas and Children's Medical Center of Dallas	Major role in the acquisition of data; study concept or design; and analysis or interpretation of data
Daniel A. Freedman, DO	Department of Neurology, Dell Medical School, Austin, TX	Study concept or design
Dara V.F. Albert, DO, MEd	Division of Child Neurology, Nationwide Children's Hospital, Columbus, OH	Study concept or design; analysis or interpretation of data

## Appendix 2 Coinvestigators

**Table:**

Name	Location	Role	Contribution
Afsaneh Talai, MD	Division of Pediatric Neurology, University of Texas Southwestern Medical Center and Children's Medical Center of Dallas	Lead author/coinvestigator	Survey development/distribution, writing paper
Daniel A. Freedman	The University of Texas at Austin Dell Medical School, Austin	Author/coinvestigator	Survey development/distribution, writing paper
Dara V.F. Albert	Division of Neurology, Nationwide Children's Hospital/The Ohio State University, Columbus	Author/principal-investigator	Survey development/distribution, writing paper
Elizabeth Fenstermacher	Children's Hospital Colorado, Aurora	Coinvestigator	In study group, no direct involvement in project
Gisela Maria Sandoval	Stanford Medicine, CA	Coinvestigator	In study group, no direct involvement in project
Hillary Kimbley	Children's Medical Center of Dallas, TX	Coinvestigator	In study group, no direct involvement in project
Jennifer Cohen	Connecticut Children's	Coinvestigator	In study group, no direct involvement in project
Kristen Trott	Division of Neurology, Nationwide Children's Hospital/The Ohio State University, Columbus	Coinvestigator	In study group, no direct involvement in project
Maija Steenar	Children's Hospital of Orange County	Coinvestigator	In study group, no direct involvement in project
Priya Tatachar	Ann & Robert H. Lurie Children's Hospital of Chicago, IL	Coinvestigator	In study group, no direct involvement in project
Sigita Plioplys	Ann & Robert H. Lurie Children's Hospital of Chicago, IL	Coinvestigator	In study group, no direct involvement in project
Susan Koh	Children's Hospital Colorado, Aurora	Coinvestigator	In study group, no direct involvement in project
Tracee Ridley-Pryor	Le Bonheur Children's Hospital	Coinvestigator	In study group, no direct involvement in project
Jeannette Iskander	Akron Children's	Coinvestigator	In study group, no direct involvement in project

## Glossary

ACGME: Accreditation Council for Graduate Medical Education
AES: American Epilepsy Society
ES: epileptic seizure
FND: functional neurologic disorder
FNDS: Functional Neurological Disorders Society
ILAE: International League Against Epilepsy
OSCE: Objective Structured Clinical Examination
PD: program director
PERC: Pediatric Epilepsy Research Consortium
PGY: postgraduate year
PNES: psychogenic nonepileptic seizure
UTSW: University of Texas Southwestern
